# The Relationship between Injury and Socioeconomic Status in Reference to the Fourth Korean National Health and Nutrition Examination Survey

**DOI:** 10.1186/2052-4374-26-1

**Published:** 2014-01-03

**Authors:** Sung-Kyung Kim, Hyocher Kim, Kyungsuk Lee, Hee-Tae Kang, Sung-Soo Oh, Sang Baek Ko

**Affiliations:** 1 Department of Occupational and Environmental Medicine, Wonju Severance Christian’s Hospital, Yonsei University, Wonju, Korea; 2 National Academy of Agricultural Science (NAAS), Rural Development Administration (RDA), Seoul, Suwon, Korea

**Keywords:** Injury, Socioeconomic status, Income, Occupation, Education

## Abstract

**Objectives:**

This study aims to investigate the relationship between the total injury experience rate and socioeconomic status based on the fourth Korea National Health and Nutrition Examination Survey (KNHANES).

**Methods:**

By analyzing data from the fourth KNHANES conducted from 2007 to 2009, we estimated the injury experience rate according to socioeconomic status, including the occupational characteristics of 11,837 subjects. Setting the injury experience rate as a dependent variable and socioeconomic status as an independent variable, we performed logistic regression to calculate odds ratios reflecting the likelihood of injury according to socioeconomic status while controlling for relevant covariates.

**Results:**

In 797 subjects who had injury experience over the past 1 year, 290 persons (36.4%) had a work-related injury. As their income, home value, and educational status increased, their injury experiences decreased. Among occupational groups, the craft, equipment, machine operating, and assembling workers showed the highest rate (10.6%) of injury experience, and the lowest rate (5.7%) was found in the unemployed group. After adjusting for the confounding variables, the experience of injury was significantly related to several socioeconomic factors: high income (OR = 0.54; 95% CI: 0.34-0.86), high home value (OR = 0.65; 95% CI: 0.43-0.96), low education status (OR = 1.28; 95% CI: 1.07-1.52), and specific occupations such as craft, equipment, machine operating, and assembling work (OR = 1.99; 95% CI: 1.60-2.47), skilled agriculture, forestry and fishery work (OR = 1.43; 95% CI: 1.02-2.01), and simple labor (OR = 1.38; 95% CI: 1.04-1.82).

**Conclusions:**

The injury experience rate differed depending on the socioeconomic status. A negative correlation was found between the injury experience rate and income, low home value, and education level. Moreover, a higher rate of injury experience was found in occupation groups and physical worker groups in comparison to the unemployed group and white-collar worker groups. This study would be useful in selecting appropriate priorities for injury management in Korea.

## Introduction

Injury is an important health issue [1,2]. Injury is a major contributor to morbidity, disability, and even early mortality; unexpected death that shortens life expectancy further results in increased social loss and economic costs [3]. According to a report from the World Health Organization, about 5 million people, 83.7 per 100,000, worldwide died due to injuries in 2000. This accounts for 9% of global mortality, and the cost for treating them totaled about 12% of all medical costs worldwide [4]. Thus despite the major health impact of injury, it has drawn relatively little attention among health-related concerns [5]. The situation is particularly serious in the Republic of Korea. According to OECD health data published in 2007, Korea had the second highest rate of injury associated fatalities (67.5 per 100,000) among OECD countries in 2004. This number accounted for 12.4% of all mortalities in Korea that year, and this proportion was the highest among OECD nations [6]. As injury mechanisms have been analyzed and comprehended, perceptions about injury have now shifted from seeing them as the consequences of random and unavoidable accidents to preventable accidents in most cases. In accordance with this changed appreciation about injury, several countries have been collecting data related to injuries for analysis purposes in order to develop effective policies to prevent further injuries [7].

Injuries can be categorized into fatal and nonfatal injuries. A fatal injury is defined as death resulting from the combination of all injuries sustained, and a nonfatal injury is an acute impact, short of death, on one’s health from an external force or agent [8]. The causes of both fatal and nonfatal injuries are known to vary by age and sex [8]; exposure to mechanochemical agents, unsafe work environment or activities [9,10], and stress and individual physiological characteristics [10-12] have been noted to be sources of injury. In addition, social surroundings, including physical and socioeconomic factors, are also considered to contribute to injury [13]. Haddon’s matrix describes injury as the result of interaction among a person, a causative agent, and the external environment [14]. Socioeconomic status represents the relative social position of an individual or a group that is determined by income, occupation, assets, level of education, and similar factors, and it is known to affect both individuals and external environmental factors within Haddon’s matrix [15,16]. Thus, the importance of socioeconomic factors to injury must not be underestimated. Although injury is related to level of income, occupation type, job status, and other socioeconomic factors, a limited number of studies in Korea have dealt with injury in relation to socioeconomic status. One study reported a higher incidence of injury in groups with low education levels, certain vocations that are vulnerable to injury, and low income [17,18]. Another study reported that people with higher income levels and city-dwellers had a significantly smaller proportion of injury than those with a low income or who resided in rural areas, respectively, within a given population group [7].

This study is based on data from the fourth Korea National Health and Nutrition Examination Survey (KNHANES), which contains questions for identifying the incidence of injury and accidents in workers, along with their socioeconomic and occupational characteristics. We analyzed the data to identify various characteristics of injury according to socioeconomic status and to confirm the relationship between the incidence of injury and socioeconomic level.

## Materials and methods

### Study subjects

The fourth KNHANES was conducted for 3 years from 2007 to 2009, and 23,632 (74.5%) out of 31,705 individuals sampled participated in the study [19]. Among them, 11,837 people between 20 and 59 years old who reported having sustained an injury within the past 1 year were selected for final analysis.

### Study methods

This study defined personal injury as experience of injury within the past 1 year at the time of answering the KNHANES questionnaire. Persons who reported having sustained an injury within the past 1 year were stated to have an experience of injury, and among them, injuries originating from working were noted as occupational injuries [20].

The population characteristics of age, sex, and marital status were obtained, and information on socioeconomic characteristics such as annual household income, home value, and educational level, occupation, type of employment, national basic livelihood security status, and job status were also collected. We organized the occupations into 7 groups according to the major categorizations of the 6th Korean Standard Classification of Occupations: ‘manager, professional, and administrator’, ‘clerk’, ‘service and sales worker’, ‘skilled agricultural, forestry, and fishery worker’, ‘craft, equipment, machine operating, and assembling worker’, ‘elementary worker’, and ‘unemployed’. For the type of employment, they were divided into salaried workers, self-employed, and unpaid family workers. The salaried workers were further divided into fully employed, temporary, and day workers. Finally, the type was also divided into full-time and part-time jobs according to time spent at work.

In this study, the proportions of people with injury experience among all questionnaire responders were calculated in accordance with each of the following socioeconomic factors: annual household income, type of employment, sex, age, level of education, national basic livelihood security status, home ownership, home value, and marital status. Experience with injury was set as a dependent variable, and annual household income, home ownership, education level, and type of occupation were designated as independent variables. Age, sex, and marital status were adjusted as confounding variables, and multiple logistic regression analysis was performed to compute the adjusted odds ratio and 95% confidence interval of the sample group. Statistical evaluation was done with SAS software version 9.2 (SAS Inc., Cary, NC, USA), and statistical significance was determined to be below 0.05.

## Results

### Socioeconomic and general demographic characteristics of study subjects

There were 5,047 males (42.6%) and 6,790 females (57.4%). Distribution by age groups showed that 2,097 subjects (17.7%) were in their 20s, 3,470 (29.3%) in their 30s, 3,386 (28.6%) in their 40s, and 2,884 (24.4%) in their 50s. With regard to marital status, 2,134 subjects (18.1%) stated that they had never been married, while 9,018 (76.5%) subjects reported being married; 244 subjects (2.1%) reported being bereaved, and 400 subjects (3.4%), divorced. Among the subjects, 225 (2.0%) had an annual household income of less than 5 million won (1 million Korean won is approximately US$1,000), and this group had the smallest number of subjects. The group with an annual household income between 20 million and 50 million won had the largest number of members, 5,711 (51.8%). Those who were on national basic livelihood security numbered 323 subjects (2.7%), while 11472 subjects (97.6%) were covered by National Health Insurance. There were 3,899 people (33.0%) who did not own a house, 6,708 (56.8%) owned a house, and 1,203 subjects (10.2%) owned two or more houses. Among home values, the largest number of subjects, 2,120 (28.2%), owned homes ranging between 100 million and 200 million won (approximately $US100,000-200,000). The group with a middle school diploma had the smallest number of subjects, 1,282 (10.8%), while the largest number of subjects, 5,124 (43.2%), had a high school diploma but no additional degrees. There were 610 subjects (5.5%) in the ‘skilled agricultural, forestry, and fishery worker’ occupational group, which had the fewest subjects. The service and sales worker group had the largest number of subjects, 1,886 (17.1%). The unemployed group contained 3,163 subjects (28.8%). When asked about type of employment, 5,064 subjects (64.1%) responded that they were salaried workers, while 2,384 people (30.2%) were self-employed. When the salaried workers were further subdivided, there were 845 subjects (16.8%) who listed their jobs as temporary, and 547 subjects (10.9%) reporting being day workers. Full-time workers numbered 4,216 (84.2%), and there were 792 part-time workers (15.8%) (Table 1).

**Table 1:** Demographic and socioeconomic characteristics of the subjects

**Variables**	**Categories**	**Number (%)**
Sex	Male	5,047 (42.6)
	Female	6,790 (57.4)
Age (years old)	20-29	2,097 (17.7)
	30-39	3,470 (29.3)
	40-49	3,386 (28.6)
	50-59	2,884 (24.4)
Marital status	Married	9,018 (76.5)
	Bereaved	244 (2.1)
	Divorced	400 (3.4)
	Never married	2,134 (18.1)
Household income per year	<5	225 (2.0)
(1 million KRW)	5 - < 10	586 (5.3)
	10 - < 20	1,795 (16.3)
	20 - < 50	5,711 (51.8)
	≥ 50	2,712 (24.6)
National basic livelihood security	Current recipient	323 (2.7)
	Former recipient	284 (2.4)
	Non-recipient	11,203 (94.9)
Health insurance	National health insurance	11,472 (97.6)
	Medical aid	279 (2.4)
Home ownership	No home	3,899 (33.0)
	1 home	6,708 (56.8)
	More than 1 home	1,203 (10.2)
Home value (x 1 million KRW)	< 50	925 (12.3)
	50 - < 100	1,494 (19.9)
	100 - < 200	2,120 (28.2)
	200 - < 500	2,084 (27.7)
	≥ 500	898 (11.9)
Education	≤ Elementary	1,382 (11.7)
	Middle	1,282 (10.8)
	High	5,124 (43.2)
	≥University	4,041 (34.2)
Occupation	Manager, professional, and administrators	1,800 (16.4)
	Clerks	1,179 (10.7)
	Service and sales workers	1,886 (17.1)
	Skilled agricultural, forestry, and fishery workers	610 (5.5)
	Craft, equipment, machine operating, and assembling workers	1,413 (12.8)
	Elementary workers	950 (8.6)
	Unemployed^†^	3,163 (28.8)
Employment type	Salaried workers	5,064 (64.1)
	Self-employed	2,384 (30.2)
	Family business without pay	456 (5.8)
Status of salaried workers	Fully employed	3,636 (72.3)
	Temporary	845 (16.8)
	Day worker	547 (10.9)
Working hours	Full-time	4,216 (84.2)
	Part-time	792 (15.8)

^†^including homemakers and students.

Injury Characteristics There were 797 subjects (6.7%) who had sustained an injury in the previous 1 year at the time of answering the questionnaire. Among them, 290 subjects (36.4%) incurred their injuries while working. When the injury mechanisms were studied in all of the injured persons, transportation accidents accounted for the most subjects, 296 (37.4%). The remaining injuries were 150 slips (19.0%), 115 collisions (14.5%), 76 falls (9.6%), 38 stabs/amputations (4.8%), 33 cases of intoxication (4.2%), 21 machinery accidents (2.7%), 15 lacerations (1.9%), and 8 burn wounds (1.0%).

The subjects were also classified according to the type of health care facility where they had received their initial treatment. The emergency room was visited by 170 (21.8%), and 356 subjects (45.6%) underwent treatment at an outpatient clinic. Those who were admitted to a hospital due to their injuries numbered 255 (32.7%). There were 722 subjects (99%) whose injuries were non-intentional accidents. Self-injuries with the intention to self-harm were noted in 5 subjects (0.6%), and injuries incurred due to violence from others were reported by 3 people (0.4%) (Table 2).

**Table 2:** Characteristics of injury

**Variables**	**Categories**	**Number (%)**
Any injury	No	11,040 (93.3)
	Yes	797 (6.7)
Occupational injury	No	11,547 (97.6)
	Yes	290 (2.4)
Proportion of occupational injuries among all injuries	No	507 (63.6)
	Yes	290 (36.4)
Nature of injury	Transport accident	296 (37.4)
	Fall	76 (9.6)
	Slip	150 (19.0)
	Collision	115 (14.5)
	Laceration	15 (1.9)
	Stab/amputation	38 (4.8)
	Machinery accident	21 (2.7)
	Burn	8 (1.0)
	Poisoning	33 (4.2)
	Others	39 (4.9)
Treatment type	Emergency room	170 (21.8)
	Ambulatory care	356 (45.6)
	Hospital admission	255 (32.7)
Injury intention	Unexpected accident	772 (99.0)
	Intentional self-injury	5 (0.6)
	Violence by other people	3 (0.4)

### Injury incidence according to socioeconomic level and general demographic characteristics

The males (8.4%) had a higher incidence of injury than the females (5.5%). Those in their 20s (7.4%) and 50s (7.4%) showed a higher incidence of injury experience than people in their 30s (6.2%) and 40s (6.3%). Those who reported being married (6.4%) showed a lower injury incidence than divorced subjects (10.5%). Among the subjects who earned an annual income of less than 5 million won, 10.7% of the subjects had experienced an injury, which was the highest incidence among the income levels. The incidence became smaller with increasing annual income. Although there was no statistical significance, those on national basic livelihood security had a slightly higher injury incidence (8.1%) than those not using this support program. Those on Medical Aid had a higher injury incidence (7.9%) than those on National Health Insurance. The group that owned one or more homes showed a lower incidence of injury (6.5%) than the group without home ownership. Subjects who owned a home valued below 50 million won reported the highest injury incidence, at 7.2%, and those whose home value was above 500 million won had a 4.7% injury incidence, showing a lower risk of injury in people with a higher home value. People who had an educational level of elementary school or below had an injury incidence of 8.2% showing a lower risk of injury in people with a higher educational level. When the incidence of injury was analyzed by type of employment, the ‘craft, equipment, machine operating, and assembling worker’ group had the most injured members, at 10.6%. Next was ‘skilled agricultural, forestry, and fishery workers’, among whom 7.7% were injured. ‘Elementary workers’ (7.5%) had the third highest incidence of injury. Those who reported being unemployed at the time of the survey had the lowest proportion (5.7%) of injury. Type of employment did not show any statistical significance in relation to injury incidence, but the incidence was found to be higher in salaried workers (7.4%), day workers (8.0%), and full-time workers (7.5%) (Table 3).

**Table 3:** Number of subjects who experienced injury by socioeconomic status

**Variables**	**Categories**	**Number (%)**		p value*
		**Yes**	**No**	
Sex	Male	422 (8.4)	4,625 (91.6)	<.0001
	Female	375 (5.5)	6,415 (94.5)	
Age (year)	20-29	156 (7.4)	1,941 (92.6)	0.100
	30-39	215 (6.2)	3,255 (93.8)	
	40-49	213 (6.3)	3,173 (93.7)	
	50-59	213 (7.4)	2,671 (92.6)	
Marital status	Married	580 (6.4)	8,438 (93.6)	0.008
	Bereaved	19 (7.8)	225 (92.2)	
	Divorces	42 (10.5)	358 (89.5)	
	Never married	156 (7.3)	1,978 (92.7)	
Household income per year	<5	24 (10.7)	201 (89.3)	0.020
(1 million KRW)	5 - < 10	52 (8.9)	534 (91.1)	
	10 - < 20	126 (7.0)	1,669 (93.0)	
	20 - < 50	385 (6.7)	5,326 (93.3)	
	≥50	167 (6.2)	2,545 (93.8)	
National basic livelihood security	Current recipient	26 (8.1)	297 (92.0)	0.619
	Former recipient	20 (7.0)	264 (93.0)	
	Non-recipient	750 (6.7)	10,453 (93.3)	
Health insurance	National health insurance	771 (6.7)	10,701 (93.3)	0.444
	Medical aid	22 (7.9)	257 (92.1)	
Home ownership	No	279 (7.2)	3,620 (92.8)	0.206
	Yes	517 (6.5)	7,394 (93.5)	
Home value (1 million KRW)	<50	67 (7.2)	858 (92.8)	0.047
	50 - < 100	116 (7.8)	1,378 (92.2)	
	100 - < 200	139 (6.6)	1,981 (93.4)	
	200 - < 500	131 (6.3)	1,953 (93.7)	
	≥500	42 (4.7)	856 (95.3)	
Education	≤Elementary	114 (8.2)	1,268 (91.8)	0.004
	Middle	96 (7.5)	1,186 (92.5)	
	High	355 (6.9)	4,769 (93.1)	
	≥University	230 (5.7)	3,811 (94.3)	
Occupation	Manager, professional, and administrators	108 (6.0)	1,692 (94.0)	<.0001
	Clerks	79 (6.7)	1,100 (93.3)	
	Service and sales workers	114 (6.1)	1,772 (93.9)	
	Skilled agricultural, forestry, and fishery workers	47 (7.7)	563 (92.3)	
	Craft, equipment, machine operating, and assembling workers	150 (10.6)	1,263 (89.4)	
	Elementary workers	71 (7.5)	879 (92.5)	
	Unemployed†	194 (5.7)	2,969 (94.3)	
Employment type	Salaried workers	376 (7.4)	4,688 (92.6)	0.232
	Self-employed	175 (7.3)	2,209 (92.7)	
	Family business without pay	24 (5.3)	432 (94.7)	
Status of salaried workers	Fully employed	275 (7.6)	3,361 (92.4)	0.423
	Temporary	54 (6.4)	791 (93.6)	
	Day workers	44 (8.0)	503 (92.0)	
Working hours	Full-time	317 (7.5)	3,899 (92.5)	0.414
	Part-time	53 (6.7)	739 (93.3)	

*p value by chi-squared test.

†including homemakers and students.

### Rates of emergency room visits and hospital admissions in terms of income level, home value, education, and type of job

Although no statistical significance was shown, those with a lower income level had a higher frequency of visits to an outpatient clinic or emergency room when injured. In the group with an annual household income of less than 5 million won, 5.8% had visited an outpatient clinic or emergency room, and 4.3% of the group with an annual income of more than 50 million won had paid a visit to an outpatient clinic or emergency room. According to the value of one’s home, no statistical difference was found among the groups, but the incidence was lowest in the group with a home valued at more than 500 million won (3.2%). Furthermore, people with a lower educational level had injuries that required medical attention in an outpatient setting or emergency room. Specifically, among the people with an elementary school education or less, 5.2% visited a medical institution for their injuries, and among those with a university education or higher, the visit rate was 3.8%. By type of occupation, ‘craft, equipment, machine operating, and assembling workers’ had the highest incidence of injury that required professional medical assistance at a clinic or emergency room, at 6.7%; ‘elementary workers’ had an incidence of 5.4% and ‘skilled agricultural, forestry, fishery workers’ had an incidence of 5.3%. The ‘manager, professional, and administrator’ group and unemployed group each had an incidence of 4.1% requiring medical assistance (Table 4).

**Table 4:** Rate of emergency room or ambulatory care visits among those who experienced injury

**Variables**	**Categories**	**Number (%)**		p value*
		**Yes**	**No**	
Household income per year	<5	13 (5.8)	212 (94.2)	0.1801
(1 million KRW)	5 - < 10	38 (6.5)	548 (93.5)	
	10 - < 20	79 (4.4)	1716 (95.6)	
	20 - < 50	257 (4.5)	5454 (95.5)	
	≥50	117 (4.3)	2595 (95.7)	
Home value	<50	40 (4.3)	885 (95.7)	0.474
(1 million KRW)	50 - < 100	71 (4.5)	1,423 (95.5)	
	100 - < 200	96 (4.8)	2,024 (95.3)	
	200 - < 500	94 (4.5)	1,990 (95.5)	
	≥500	29 (3.2)	869 (96.8)	
Education	≤Elementary	72 (5.2)	1,310 (94.8)	0.0353
	Middle	70 (5.5)	1,212 (94.5)	
	High	234 (4.6)	4,890 (95.4)	
	≥University	155 (3.8)	3,886 (96.2)	
Occupation	Manager, professional, and administrators	73 (4.1)	1,727 (95.9)	0.0014
	Clerks	57 (4.8)	1,122 (95.2)	
	Service and sales workers	85 (4.5)	1,801 (95.5)	
	Skilled agricultural, forestry, and fishery workers	32 (5.3)	578 (94.8)	
	Craft, equipment, machine operating, and assembling workers	95 (6.7)	1,318 (93.3)	
	Elementary workers	51 (5.4)	899 (94.6)	
	Unemployed	121 (3.8)	3,042 (96.2)	

*p value by chi-squared test.

Those with a lower income level also had a higher hospital admission rate. The group earning less than 5 million won had an admission rate of 5.8%, while the rate was 4.3% for the group with an annual income of more than 5 million won. The group with a home value of more than 500 million won showed the lowest rate (1.5%) of admission, but the difference by home value was not statistically significant. Regarding education, people with an elementary school diploma or less had a 3.0% admission rate, which was significantly higher than the 1.8% admission rate found in the group with a university diploma or higher. The ‘craft, equipment, machine operating and assembling worker’ group had the highest admission rate, at 3.9%, and next was the ‘skilled agricultural, forestry, and fishery workers’, who had a 2.8% admission rate. The admission rate for the service and sales group was 1.6% (Table 5).

**Table 5:** Rate of hospitalizing for injury among those who experienced an injury

**Variables**	**Categories**	**Number (%)**		p value*
		**Yes**	**No**	
Household income per year (1 million KRW)	<5	13 (5.8)	212 (94.2)	0.1194
	5 - < 10	38 (6.5)	548 (93.5)	
	10 - < 20	79 (4.4)	1716 (95.6)	
	20 - < 50	257 (4.5)	5454 (95.5)	
	≥50	117 (4.3)	2595 (95.7)	
Home value	<50	24 (2.6)	901 (97.4)	0.065
(1 million KRW)	50 - < 100	44 (3.0)	1,450 (97.1)	
	100 - < 200	42 (2.0)	2,078 (98.0)	
	200 - < 500	38 (1.8)	2,046 (98.2)	
	≥500	13 (1.5)	885 (98.6)	
Education	≤Elementary	41 (3.0)	1,341 (97.0)	0.0382
	Middle	25 (2.0)	1,257 (98.1)	
	High	126 (2.5)	4,998 (97.5)	
	≥University	73 (1.8)	3,968 (98.2)	
Occupation	Manager, professional, and administrators	33 (1.8)	1,767 (98.2)	0.0006
	Clerks	21 (1.8)	1,158 (98.2)	
	Service and sales workers	31 (1.6)	1,855 (98.4)	
	Skilled agricultural, forestry, and fishery workers	17 (2.8)	593 (97.2)	
	Craft, equipment, machine operating, and assembling workers	55 (3.9)	1,358 (96.1)	
	Elementary workers	20 (2.1)	930 (97.9)	
	Unemployed	73 (2.3)	3,090 (97.7)	

*p value by chi-squared test.

### Relation of injury to income level, home value, education, and type of occupation

After multiple logistic regression analysis, the odds ratio between the injury incidence and income groups decreased with increasing annual income. The odds ratios for the groups with an income between 20 million and 50 million won and an income of 50 million won or more relative to the group with an annual income of 5 million won or less were 0.6 (95% C.I.: 0.38-0.93) and 0.54 (95% C.I.: 0.34-0.86), respectively. According to an analysis by home value, the odds ratio became smaller as the home value increased above the reference group with homes valued at less than 50 million won. However, the reduction in odds ratios did not have statistical significance except for the group with a home value of more than 500 million won (OR: 0.65, 95% C.I.: 0.43-0.96). With a low level of education, the injury odds ratio increased. When compared to people with an educational level of university or higher, those who had graduated from high school or below showed a 1.28 times (95% C.I.: 1.07-1.52) higher risk of injury, and the odd ratios for the groups with a middle school diploma and with an elementary school diploma were 1.57 (95% C.I.: 1.20-2.05) and 1.89 (95% C.I.: 1.44-2.48), respectively. By type of job category, the ‘craft, equipment, machine operating, and assembling workers’ had an odds ratio of 1.99 (95% C.I.: 1.60-2.47) relative to the unemployed individuals. ‘Skilled agricultural, forestry, and fishery workers’ had an odds ratio of 1.43 (95% C.I.: 1.02-2.01), and ‘elementary workers’ had an odds ratio of 1.38 (95% C.I.: 1.04-1.82) (Table 6).

**Table 6:** Odds ratios and 95% confidence intervals for socioeconomic factors affecting the injury experience rate by multiple logistic regression analysis

**Variables**	**Categories**	Crude OR*	**95% CI†**	**Adjusted OR‡**	**95% CI**
Household income per year (1 million KRW)	<5	1		1	
	5 – < 10	0.81	0.49–1.35	0.82	0.49–1.38
	10 – < 20	0.63	0.39–0.99	0.64	0.40–1.02
	20 – < 50	0.6	0.39–0.93	0.6	0.38–0.93
	≥50	0.55	0.35–0.86	0.54	0.34–0.86
Home value	<50	1		1	
(1 million KRW)	50 – < 100	1.08	0.79–1.47	1.11	0.81–1.52
	100 – < 200	0.9	0.66–1.22	0.92	0.68–1.25
	200 – < 500	0.86	0.63–1.17	0.89	0.65–1.21
	≥500	0.63	0.42–0.94	0.65	0.43–0.96
Education	≥University	1		1	
	≤Elementary	1.49	1.18–1.88	1.89	1.44–2.48
	Middle	1.34	1.04–1.71	1.57	1.20–2.05
	High	1.23	1.03–1.46	1.28	1.07–1.52
Occupation	Unemployed†	1		1	
	Manager, professional, and administrators	1.05	0.83–1.34	1.05	0.83–1.33
	Clerks	1.19	0.91–1.55	1.18	0.90–1.54
	Service and sales workers	1.07	0.85–1.35	1.08	0.86–1.36
	Skilled agricultural, forestry, and fishery workers	1.38	1.01–1.91	1.43	1.02–2.01
	Craft, equipment, machine operating, and assembling workers	1.96	1.58–2.44	1.99	1.60–2.47
	Elementary workers	1.35	1.02–1.78	1.38	1.04–1.82

*odds ratio, †confidence interval.

‡: adjusted for age, sex, and marital status.

## Discussion

This study determined the total occurrence of injuries in the general population of Korea and the incidence of occupationally related injury using answers related to injury from the KNHANES questionnaire and analyzed its association with socioeconomic factors. The proportion of subjects who sustained at least 1 injury in the 1-year period previous to the survey was 6.7%, and among the injured subjects, 2.4% were work-related injuries. Occupational injury accounted for 36.4% of all the reported injuries. In the Korean Working Conditions Survey sponsored by the Korean Department of Labor in 2006, 2010, and 2011, the average injury rate was 2.4%. The rate of occupational injury was 2%, and workplace injuries accounted for 84% of all the injuries received. A similar survey of 27 EU nations in 2005 revealed a mean injury rate of 9.1% (3.7%-21.5%) [21]. In the present study, the occupational injury rate was 2.4%, which was similar to the findings of the Korean Working Conditions Survey, but lower than the European average.

In previous studies, it was found that each increased level of socioeconomic status shows a better health status than the level below it in a dose–response relationship [22], and this socioeconomic gradient is evident in both fatal [23-34] and non-fatal injuries [26,28,35-40]. We found the same in our study. As the levels of income and education increased, the injury incidence was reduced. The group with an annual income of more than 50 million won had an odds ratio of 0.54 (95% C.I.: 0.34-0.86) relative to the group with an income of less than 5 million won, and the group with a home valued at more than 500 million won had an odds ratio of 0.65 (95% C.I.: 0.43-0.96) compared to the group with a home valued at less than 50 million won. People with an elementary school education or below had an odds ratio of 1.89 (95% C.I.: 1.44-2.48) relative to those with a university or higher education. The injury rates that required a visit to an outpatient clinic and emergency room (Table 5) and that needed hospital admission were also higher in vulnerable socioeconomic groups who earned low income, had less education, owned a less expensive home, and worked in elementary labor. The results of our study were in accord with 2 previous studies.

The type of occupation and job status can also be factored in to consider the socioeconomic status [17]. The ‘craft, equipment, machine operating, and assembling worker’ group had the highest injury incidence of 10.6% among all of the occupational groups. ‘Skilled agricultural, forestry, and fishery worker’ (7.7%), ‘elementary worker’ (7.5%), ‘service and sales worker’ (6.1%), ‘clerk’ (6.7%), and ‘managerial and professional worker’ (6.0%) groups followed in that order, revealing concurrence with previous study results showing that manual labor has a higher risk of injury [24,26,31,36]. The unemployed had a significantly lower injury incidence than those with jobs, and their rate of seeking medical attention from an outpatient clinic or emergency room was also lower than those of the other groups. In addition, the unemployed group reported lower odds of injury than the other groups, and the group showed a particularly significant reduction in the odds of injury compared to ‘skilled agricultural, forestry, and fishery worker’, ‘craft, equipment, machine operating, and assembling worker’, and ‘elementary worker’ groups. This information contradicts previous studies reporting slightly but not significantly higher injury odds of 1.03 in the jobless than in those who were employed [17]. Our finding provides evidence that having a job may elevate injury risk. Our study also noted the smallest total number of injuries (5.7%) and injury risk in all subjects in the unemployed group, but the hospital admission rate, which reflects the severity of injury, was 2.3%, which was higher than the rate found in the white-collar worker group. This is in accord with a previous study that found the risk of mortality for jobless people was 2.26 times higher than that of other groups [17]. The reason for the higher risk can be explained by reports that noted a relationship with higher incidences of violent crime [30] and suicide [25].

Socioeconomic status is known to affect injury risk through a complex process. The causes of injuries originating from socioeconomic discrimination are psychosocial factors such as stress from poverty and intentional self-inflicted injury due to the stress from inequality as a result, raising the quality of material factors such as providing a livable income and adequate housing is effective in lowering the injury risk [17]. Lower socioeconomic status may result from poor housing and transportation, higher crime rates, and underemployment [26,41-43]. In addition to physical factors, there are occupation-related factors that also affect injury incidence [44]. Socioeconomic factors are also influenced by a geographical factor that has been interpreted as the degree of accessibility to various appropriate public services such as secure public safety measures, safe road, and recreational area. Also, a prosperous town has low crime rate. It has been suggested that the low crime rate is due to limiting strangers from accessing the town and encouraging good behavior among members of the community, and these positive activities have some protective value in preventing injury [11]. Those with a low socioeconomic level are likely to neglect the fact that injury is preventable, and this inattention may function as a factor that leads to the potential for injury [45]. A parent’s socioeconomic status is also an important factor in injury risk for children. Although the effect varies by the age and sex of a child, a parent’s efforts in providing their children with appropriate education, housing, and injury preventative measures are presently considered imperative for reducing injury risk [46-52].

There present some limitations for this study. This study’s variables are obtained from a questionnaire and interview, and it is possible that the study is biased with recall bias from subjects. During the survey, surveyors tried to visit each subjects to obtain data directly. Data was not available from those who were admitted to a hospital or had deceased, and data from these subjects may be left out for interpretation. However, possibility of selection bias that may underestimates injury incidence is low, because fourth KNHANES was carried out as an individualized interview to each subject without knowing subjects’ trauma histories. Future studies further need more in-depth interviews and objective reviews on data to improve the analysis, and it is also considered that supplementing questions related injury, training surveyors, standardization of indicators, and checking reliability and validity of indicators are necessary to refine the outcomes in future studies. In this study, only a person’s income was taken in for socioeconomic evaluation, but the future studies need to consider obtaining more information on a person’s asset including car and financial asset for multidimensional interpretation. Lastly, there present a limitation to examine causal relationship between socioeconomic state and injury in this study, because it was a cross-section study. In order to investigate the causal relationship, a prospective cohort study is required.

## Conclusions

Despite these limitations, by using the answers to the injury-related questions of the fourth KNHANES, this study was able to confirm the differences in injury incidence among various socioeconomic levels represented by differing education levels, incomes, home values, and types of occupations. Furthermore, the authors investigated the relationship between socioeconomic status and injury and confirmed that lower income and education levels were associated with a statistically significant increase in the odds of injury. In addition, the groups with jobs had a higher risk of injury than the unemployed group, and manual laborers had significantly elevated injury risk. Through this study, it was determined that many injuries have preventable aspects, and vulnerable socioeconomic groups were identified as being an important focus for injury risk management. Information on the frequency of injury and conditions inciting injury enables the recognition of injury severity and characteristics and also provides a basis for the development of injury prevention and management policy [2]. A gradual multistage approach to injury-related problems and the selection of groups who need priority intervention are necessary to reduce injury incidence [7]. Developing interventions will require identifying the present state of injury, followed by recognizing causes and factors that are associated with injury. This information can be used to work out a basis for the distribution of available resources to prevent and manage injuries. We believe that the results of this study can be used as a reference for selection of a group in Korea that requires priority in receiving intervention.

## Competing interests

The authors declared that they have no competing interests.

## Authors’ contributions

SKK and SBK designed a research method, analyzed the data and drafted the manuscript. HK, KL, HTK, and SSO participated in research process and collected data. All authors read and approved the final manuscript.
